# Formation of a Double Diamond Cubic Phase by Thermotropic Liquid Crystalline Self‐Assembly of Bundled Bolaamphiphiles

**DOI:** 10.1002/anie.201602734

**Published:** 2016-06-07

**Authors:** Xiangbing Zeng, Marko Prehm, Goran Ungar, Carsten Tschierske, Feng Liu

**Affiliations:** ^1^ Department of Materials Science and EngineeringUniversity of SheffieldSheffieldS1 3JDUK; ^2^ Institute of ChemistryMartin Luther University, Halle-Wittenberg06120HalleGermany; ^3^ Department of PhysicsZhejiang Sci-Tech University310018HangzhouP.R. China; ^4^ State Key Laboratory for Mechanical Behavior of MaterialsXi'an Jiaotong UniversityXi'an710049P.R. China

**Keywords:** amphiphiles, block copolymers, liquid crystals, phase transitions, self-assembly

## Abstract

A quaternary amphiphile with swallow‐tail side groups displays a new bicontinuous thermotropic cubic phase with symmetry *Pn* $\bar{3}$ *m* and formed by two interpenetrating networks where cylindrical segments are linked by H bonds at tetrahedral junctions. Each network segment contains two bundles, each containing 12 rod‐like mesogens, lying along the segment axis. This assembly leads to the first thermotropic structure of the “double diamond” type. A quantitative geometric model is proposed to explain the occurrence of this rare phase.

Among the various self‐assembled mesoscale morphologies, intriguing cubic phases are displayed by lyotropic and thermotropic^1^, ^2^, ^3^ liquid crystals (LCs) and block copolymers.^4^ There are two classes of cubic phases in thermotropic LCs: the “bicontinuous” and the “micellar” type.^5^, ^6^ Most molecules forming such phases consist of two incompatible parts, typically aromatic and aliphatic, which tend to segregate into two subphases. In bicontinuous cubic (Cub_bi_) structures, each of the two interlocked subphases form a continuum, whereas in the micellar phases one forms discrete finite domains. The two confirmed Cub_bi_ phases in thermotropic LCs are the double‐network double gyroid phase with symmetry *Ia* $\bar{3}$ *d*,^7^ and the triple‐network phase with symmetry *Im* $\bar{3}$ *m*.^8^ Although the thermotropic *Ia* $\bar{3}$ *d* phase has been observed in rod‐like molecules, especially polycatenars (rod‐like with more than one end‐chain),^3^, ^5^, ^8^, ^13^ in some taper‐shaped compounds,^9^, ^10^, ^11^ and, recently, in bolaamphiphiles,^12^ the *Im* $\bar{3}$ *m* phase has so far only been found in polycatenars.^3^, ^8^, ^13^ The polycatenar aromatic rod‐like cores lie approximately perpendicular to the network segments (Figure 1 a). It has been reported recently that in both the *Im* $\bar{3}$ *m* and the *Ia* $\bar{3}$ *d* phase, the direction of the rods twists along the network segment and that the match between helix pitch and rod‐length determines the observed phase type.^13^

**Figure 1:**
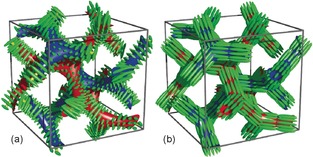
Double gyroid cubic phase in a) a polycatenar mesogen^13^ and b) a bolaamphiphile with a branched lateral chain.^12^ Blue and red indicate the two enantiomorphic networks; the green ellipsoids represent the rod‐like aromatic cores.

Although there have been sporadic accounts of other suspected Cub_bi_ phases,^14^, ^15^, ^16^, ^17^, ^18^ there has been no confirmed report of a third Cub_bi_ phase in thermotropic LCs to date.^19^ Herein we report what, to our knowledge, is the first confirmed case of a thermotropic Cub_bi_ phase with the “double diamond” (DD) structure. The structure, found in a bolamphiphile with a branched side‐chain, was determined by X‐ray diffraction (XRD). Based on quantitative modelling of the phase geometry, we also propose a rational explanation of why the DD phase occurs in the particular compound studied.

Rod‐like bolaamphiphiles with flexible side‐chains display an abundance of thermotropic LC phases, many of them of honeycomb type.^20^ As the side‐chain volume increases, lamellar, cubic, and eventually the unique axial columnar phases are displayed.^21^ The one cubic phase reported to date in these systems is of the double gyroid *Ia* $\bar{3}$ *d* type, seen in compounds **2 a** and **2 b** (Table 1).^12^ Unique to this *Ia* $\bar{3}$ *d* phase is that here the rod‐like mesogens align in bundles parallel to the segments of the two networks (Figure 1 b). The subject of the present report is compound **1**. It has a shorter aliphatic (CH_2_)_3_ spacer connecting each of the two perfluorinated segments to the branching point and the aromatic core.

**Table 1:** Molecular structure and phase‐transition temperatures of compounds **1** and **2**. 
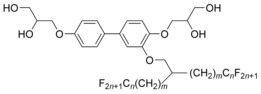

Compound	*m*	*n*	Phases and transition temperatures [°C]^[a]^
**1**	3	8	Cr 74 [*18.9*] Lam_Sm_ 130 [*4.12*] Cub/*Pn* $\bar{3}$ *m* 146 [*1.55*] Iso
**2 a**	11	8	Cr 75 Lam_Sm_ 102 Lam_N_ 106 Col_rec_/*c*2*mm* 132 Cub/*Ia* $\bar{3}$ *d* 154 Iso
**2 b**	11	10	Cr 108 Lam_Sm_ 132 Lam_N_ 133 Col_rec_/*c*2*mm* 145 Cub/*Ia* $\bar{3}$ *d* 178 Iso

[a] Values in square brackets indicate the corresponding transition enthalpy values (Δ*H*/kJ mol^−1^; determined by DSC, first heating scan, 10 K min^−1^; see Figure S1). Cr=crystalline solid; Lam_Sm_=lamellar smectic phase; Lam_N_=lamellar nematic phase; Col_rec_/*c*2*mm*=rectangular columnar phase with *c*2*mm* lattice; Cub/*Pn* $\bar{3}$ *m*=double diamond cubic phase (DD) with *Pn* $\bar{3}$ *m* lattice; Cub/*Ia* $\bar{3}$ *d*=double gyroid cubic phase with *Ia* $\bar{3}$ *d* lattice; Iso=isotropic liquid. Data for compounds **2 a** and **2 b** are from Ref. [12].

Above the crystal melting point at 74 °C, **1** has a thermodynamically stable LC region over more than 70 °C (see the differential scanning calorimetry plot (DSC) in Figure S1 in the Supporting Information). Confirming the LC nature of all mesophases, over this entire range there is only diffuse scatter in the wide‐angle XRD region (WAXS), with a maximum corresponding to a Bragg spacing of *d*=0.55 nm (Figure 2 c (inset); see also Figure S2). The polarized optical images recorded either side of the 130 °C endotherm are shown in Figure 2 a,b. The birefringent mosaic texture in Figure 2 a represents the Lam_Sm_ phase, while the black non‐birefringent image in Figure 2 b suggests a cubic phase.

**Figure 2:**
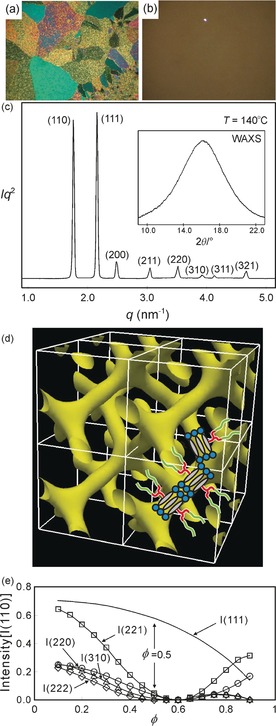
Characterization of compound **1**: a) Texture as seen between crossed polarizers at *T*=110 °C and b) *T*=135 °C. c) SAXS diffractogram of the Cub/*Pn* $\bar{3}$ *m* phase (inset: the wide‐angle curve). d) Reconstructed electron density map for the Cub/*Pn* $\bar{3}$ *m* phase (see Table S2); only low‐electron‐density regions are shown (see also Figure S6). e) Theoretical XRD intensities for the d‐surface based the *Pn* $\bar{3}$ *m* phase of the small‐angle Bragg reflections relative to the intensity of the first (110) peak as a function of the volume fraction (*ϕ*) of the “decorated” (thickened) d‐surface;^23^ Part (e) is reproduced with permission from the American Chemical Society.^23^

XRD patterns of the low‐temperature mesophase of **1** are shown in Figures S2 c, d and Figure S3, and those of the high‐temperature mesophase are shown in Figure 2 c and Figures S2 a, b (see Tables S1–2 for calculated *d* spacings). The LC phase below 130 °C is a lamellar phase having the rod‐like units oriented parallel to the layer plane (Lam_Sm_).^20^ The Lam_Sm_ phase is discussed in more detail in the Supporting Information (Figures S4 and S5). This report instead focuses on the cubic phase occurring at *T*>130 °C.

In the cubic phase, the 1/*d* ^2^ values stand in the ratio 2:3:4:6:8:10:11:14 and can be indexed on a primitive lattice (*a*_cub_=5.02 nm) with the first reflection indexed as (110) (see Figure 2 c and Table S2). The observed diffraction peaks obey the rules 0*kl*: *k*+*l*=2*n* and *h*00: *h*=2*n*. Two space groups comply with these conditions, *Pn* $\bar{3}$ (no. 201) and *Pn* $\bar{3}$ *m* (no. 224). As liquid crystals have the tendency to assume the highest symmetry, *Pn* $\bar{3}$ *m* is our first choice. Using the diffraction intensities from the powder SAXS pattern (Figure 2 c and Table S2), we reconstructed the electron density map *ρ*(*xyz*), as shown in Figure 2 d and Figure S6. The yellow isoelectron surfaces enclose approximately the aromatic cores, the glycerol, and partly the alkyl groups (low *ρ*), while the high‐density regions outside (enclosed within the blue surfaces in Figure S6) contain the R_F_ branches. The structure can be best described as consisting of two identical interwoven infinite diamond networks. Each network contains tetrahedral four‐way junctions connected by column‐like segments. A unit cell contains two junctions with point symmetry $\bar{4}$ 3*m*, both on the same network, at positions (1/4 1/4 1/4 ) and (3/4 3/4 3/4 ). The junctions of the other network are in neighboring cells. Halfway between the two networks lies the D‐type infinite periodic minimum surface,^22^ and the blue R_F_ regions in Figure S6 roughly follow that surface.

From the lattice parameter *a*_cub_, the length of the column between two junctions was calculated as 4.35 nm (1/2×3^1/2^×*a*_cub_), which is about twice the molecular length *L*=2.10 nm measured between the ends of the two glycerol units. Accordingly, it is suggested that each column consists of two consecutive bundles of rod‐like mesogens (Figure 2 d). From the total number of molecules in a cell (about 93) each bundle contains circa 12 molecules, as the unit cell is composed of four interjunction segments or eight bundles (Table S3). We note that a network segment in the skeletal *Ia* $\bar{3}$ *d* phase of compounds **2 a** and **2 b** also consist of two consecutive bundles with 12 molecules in each.^12^ As discussed in Ref. 21, the number of molecules in a bundle is limited by the fact that the side‐chains of all molecules must have access to the inter‐network continuum.

Garstecki and Holyst have calculated the relative diffraction intensities based on the D minimum surface of finite thickness as a function of that thickness, that is, its volume fraction *ϕ* (Figure 2 e).^23^ It is clear that for systems that can be approximated by a two‐level electron‐density model, which means most common LC types, the first peak, (110), is the strongest in the entire range of *ϕ* values. For compound **1** the fluorocarbon volume fraction is *ϕ*=0.5 (Table S3). According to Figure 2 e, we would therefore expect only the first two peaks, (110) and (111), to be strong, the others being significantly weaker, which is indeed the case (Figure 2 c).^19^

In Reference 8, an attempt was made to describe semiquantitatively the relationship between the architecture of the molecules and the Cub_bi_ phase they choose to adopt. Its application to the present case is shown in Figure 3. We compare the average radial distribution of volume functions *dV*/d*r* for two Cub_bi_ structures: the double gyroid *Ia* $\bar{3}$ *d* and the double diamond *Pn* $\bar{3}$ *m*, as well as for the hexagonal columnar (Col_h_) phase. Here *r* is the distance from the central axis of the segments of the network (or the column in Col_h_). For the Col_h_ phase the cylinders grow unhindered in radius, with *dV/dr* ∝ *r*, until the parallel cylinders clash. Since further growth in volume takes place only where the cylinders do not overlap, the *dV/*d*r* value drops sharply and falls to 0 when all space is filled. Thus, the ideal shape of a molecule to fit into the Col_h_ phase is one with a fan‐shape for which the cross‐sectional area (*A*) increases linearly as one moves from the column center, that is, *A*(*r*) ∝ *dV/dr* ∝ *r*, and then drops quite abruptly to 0. However, molecules whose cross‐sectional shape can be described by a fractional exponent, that is, by *A*(*r*) ∝ *dV/dr* ∝ *r* ^*m*^, with 0<*m*<1, are likely to form a Cub_bi_ phase.

**Figure 3:**
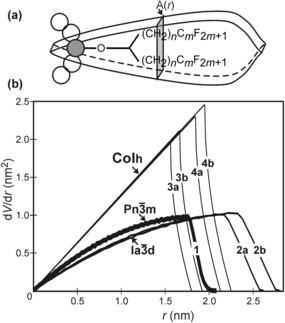
a) Envelope of a bolaamphiphile viewed along the rod‐like core (dark circle). Empty circles represent neighboring rods in the bundle. b) Radial distribution of volume functions *dV/dr* for the *Ia* $\bar{3}$ *d*, *Pn* $\bar{3}$ *m*, and Col_h_ phases. The curves plot the increase in occupied volume as the radius (*r*) of the cylindrical columns or network segments increases. Each curve is calculated (numerically for cubics) using the experimental unit‐cell volume for the respective compound (indicated), divided by the number of molecules in the cell. The areas under the curves are proportional to molecular volume. Incidentally, the shape of *dV/dr* for the double gyroid and the triple‐network *Im* $\bar{3}$ *m* phases are very similar.^8^ The molecular cross‐sectional area profile, *A*(*r*), in (a) should match *dV/dr* of a particular phase for uniform volume filling.

Considering only examples of bolaamphiphiles where the rods are coaxial with the columns,^12^, ^21^ we consider the average cross‐section normal to the column. Col_h_ phases were observed in terphenyl bolaamphiphiles **3** and **4** with two branched side‐chains (Figure 4).^21^ The two branches in compounds **3** and **4** require extra lateral space; this is what the Col_h_ phase, with its high *dV/dr* value at a larger radius *r*, conveniently provides, and the above compounds form variants of Col_h_.^21^ Their different side‐chain lengths (smallest in **3 a**, largest in **4 b**) mean different intercolumnar distances, hence different end‐points of the linear increase in *dV/dr*. On the other hand, compounds **1** and **2**, with only one branch, require less lateral space (lower *dV/dr*) at large *r* values and thus fit better into Cub_bi_ phases. Similar to **3** and **4**, compounds **2 a** and **2 b** differ somewhat in their inter‐network distances, and thus have their clash points at different *r* values.

**Figure 4:**
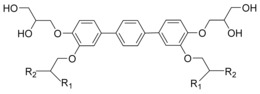
Terphenyl bolaamphiphiles **3 a**,**b** and **4 a**,**b,** forming hexagonal columnar (Col_h_) LC phases with coaxial rod bundles. **3 a**: R_1_=C_9_H_19_; R_2_=(CH_2_)_3_C_6_F_13_. **3 b**: R_1_=C_11_H_23_; R_2_=(CH_2_)_3_C_8_F_17_. **4 a**: R_1_=R_2_= (CH_2_)_11_C_4_F_9_. **4 b**: R_1_=R_2_=(CH_2_)_11_C_6_F_13_.

Figure 3 also provides a clue as to why compound **1** adopts the DD phase whereas **2 a** and **2 b** form the gyroid. The gyroid phase requires the side‐chains to be long enough to fill a relatively large distant volume (area under the *Ia* $\bar{3}$ *d* curves at large *r* values). Although this is possible for **2 a** and **2 b** with their long C_11_H_22_ spacers, it is not for compound **1**. Conveniently, however, the *Pn* $\bar{3}$ *m* DD phase does not require filling of large distant volume and generally provides less inter‐network space (less overall area under the *dV/dr* curve) that is well suited to the smaller side‐chains of compound **1**. In the DD structure, the networks are more tightly knit than in the gyroid when scaled to the same inter‐junction distance; this is clear from the simple fact that the former has four‐way and the latter three‐way junctions. The unique feature of the skeletal Cub_bi_ phases in side‐branched bolaamphiphiles is that the inter‐junction distance is fixed to *nL*, where *n* is an integer (here *n*=2). No such strict restriction applies to polycatenar or fan‐shaped compounds, which are free to adjust the number of molecules between network junctions for their end‐chains to match the internetwork volume required by the gyroid. This is possibly one reason for the apparent absence of DD phase in polycatenar and fan‐shaped compounds. Incidentally, even in block copolymers (BCPs) there seems to be no unambiguous confirmation of the existence of a DD structure,^4^ even though at the time this was claimed to be the first Cub_bi_ phase in BCPs; only in lyotropic systems is this cubic phase common.

The present work provides the first unambiguously proven thermotropic DD phase^19^ and demonstrates that new three‐dimensional LC structures can be engineered using the principle of skeletal networks of axial mesogen bundles and the additional packing constraints that it imposes. Preliminary studies suggest that other novel cubic phases may be generated in this way.

## Supporting information

As a service to our authors and readers, this journal provides supporting information supplied by the authors. Such materials are peer reviewed and may be re‐organized for online delivery, but are not copy‐edited or typeset. Technical support issues arising from supporting information (other than missing files) should be addressed to the authors.

Supplementary
