# Supplementary material for: Formation of a Double Diamond Cubic Phase by Thermotropic Liquid Crystalline Self‐Assembly of Bundled Bolaamphiphiles
Source: Angew Chem Int Ed Engl. 2016 Jun 7;55(29):8324–7. doi: 10.1002/anie.201602734 (PMC5089568; doi:10.1002/anie.201602734)
Supplement: Supplementary file 1 — Supplementary [file ANIE-55-8324-s001.pdf]

Angewandte Chemie
Eine Zeitschrift der Gesellschaft Deutscher Chemiker

Supporting Information

Formation of a Double Diamond Cubic Phase by Thermotropic Liquid Crystalline Self-Assembly of Bundled Bolaamphiphiles

Xiangbing Zeng, Marko Prehm, Goran Ungar, Carsten Tschierske,* and Feng Liu**

anie_201602734_sm_miscellaneous_information.pdf

Supporting Information

Contents

1. Syntheses and analytical data of the materials 1
    1.1 Acetonides A*n*/*m* 2
    1.2 Synthesis of compound 1 3
2. Experimental techniques 3
    2.1 DSC and optical microscopy 3
    2.2 X-ray scattering on powder-like and aligned samples 3
    2.3 Synchrotron X-ray diffraction and electron density reconstruction 3
3. Additional data 5
    3.1 DSC traces 5
    3.2 Additional XRD data 5
4. Synchrotron X-ray and structural data 6
5. Calculation of number of molecules per cell 9
6. References 9

1. Syntheses and analytical data of the materials

Unless otherwise noted, all starting materials were purchased from commercial sources and were used as obtained. Preparative thin layer chromatography was performed with a Chromatotron (Harrison-Research) using silica gel 60 PF_{254} (Merck). Column chromatography was performed with silica gel 60 (63–200 µm, Merck). Confirmation of the structures and purity of intermediates and products was obtained by NMR spectroscopy (VARIAN Unity 500,

VARIAN Gemini 200 and VARIAN VRX 400 spectrometers, all spectra were recorded at 27 °C). Microanalyses were performed using a LECO CHNS-932 or CARLO Erba-CHNO 1102 elemental analyzer. The purity of all compounds was checked by thin layer chromatography (silicagel 60 F_{254}, Merck). Hexane/EtOAc mixtures and $CHCl_3$/MeOH mixtures were used as eluent and the spots were detected by UV radiation.

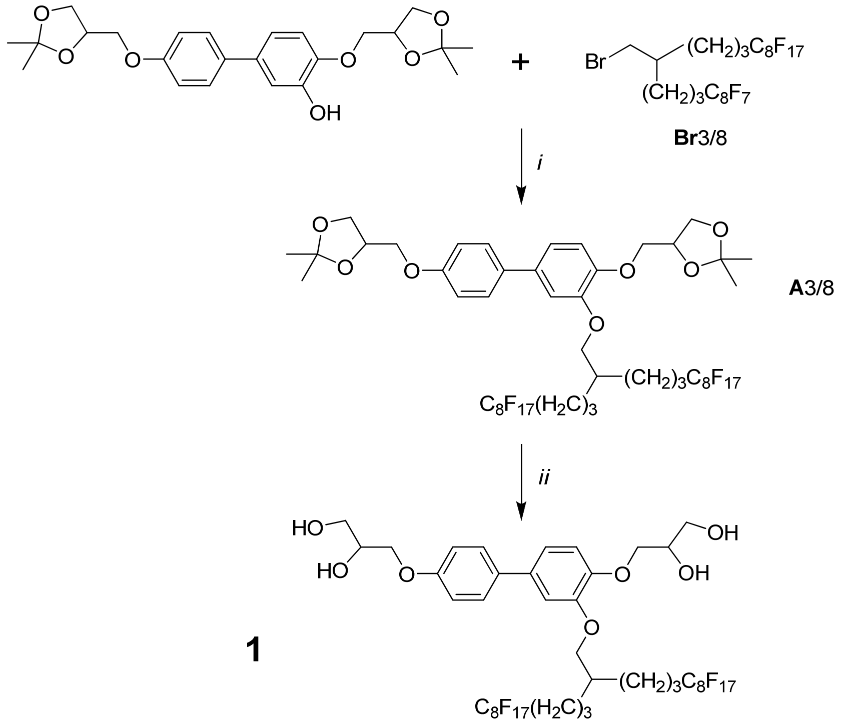

1

Scheme S1. Synthesis of compound **1**; *reagents and conditions i*: K_2CO_3, DMF, 50-60 °C; *iv*: HCl, MeOH, reflux.

1.1 Acetonides A*n*/*m*

4,4'-Bis(2,2-dimethyl-1,3-dioxolane-4-ylmethoxy)-3-[2,2-bis(4,4,5,5,6,6,7,7,8,8,9,9,10,10,11,11,11-heptadecafluoroundecyl)ethoxy]biphenyl (A3/8): A mixture of 4,4'-bis(2,2-dimethyl-1,3-dioxolane-4-ylmethoxy)-biphenyl-3-ol[S1] (100 mg, 0.23 mmol) and **Br3/8**[S2] (247 mg, 0.24 mmol), K_2CO_3 (159 mg, 1.15 mmol) and Bu_4NI (5 mg) in anhydrous DMF (50 ml) was stirred at 60 °C for 6 h. After cooling to room temperature, the reaction mixture was poured into ice-water (50 ml) and the aqueous layer was extracted with Et_2O (3x50 mL). The combined organic layers were washed with sat. aqu. LiCl water and brine. After drying over anhydrous Na_2SO_4, filtration and evaporation of the solvent, the crude product was purified by preparative thin layer chromatography (silica gel, PE/$CHCl_3$, 1:2 v/v). Colorless solid; Yield: 200 mg (64 %); mp 69-71 °C. ^{1}H-NMR ($CDCl_3$, *J*/Hz, 400 MHz): δ = 7.44 (d, ^3J(H,H) = 8.3, 2H, Ar-H), 7.09-7.03 (m, 2H, Ar-H), 6.95 (d, ^3J(H,H) = 8.2, 3H, Ar-H), 4.47 (sext, ^3J(H,H) = 5.7, 2H, OCH), 4.20-4.05 (m, 4H, OCH_2), 4.01-3.86 (m, 6H, OCH_2), 2.19-1.88 (m, 5H, CH, CH_2CF_2), 1.77-1.54 (m, 8H, CH_2), 1.46 (s, 3H, CH_3), 1.43 (s, 3H, CH_3), 1.40 (s, 3H, CH_3), 1.37 (s, 3H, CH_3). ^{19}F-NMR ($CDCl_3$, *J*/Hz, 188 MHz): δ = -81.23 (t, ^4J(F,F) = 9.9, 6F, CF_3), -114.56-(-114.65) (m, 4F, CH_2CF_2), -122.28 (s, 12F, CF_2), -123.13 (s, 4F, CF_2), -123.91 (s, 4F, CF_2), -126.51 (s, 4F, CF_2CF_3)

1.2 Synthesis of compound 1

3-{4'-(2,3-Dihydroxypropoxy)-3-[2,2-bis(4,4,5,5,6,6,7,7,8,8,9,9,10,10,11,11,11-heptadecafluoroundecyl)ethoxy]biphenyl-4-yloxy}propane-1,2-diol (1): A mixture of **A3/8** (100 mg, 0.07 mmol) and 10% HCl (5 mL) in MeOH (20 mL) was heated to reflux for 3 h. The progress of the reaction was monitored by TLC. The solvent was evaporated and the residue was dissolved in EtOAc, washed with sat. aq. $NaHCO_3$ (20 ml), water (20 ml) and brine (20 ml). After drying over anhydrous Na_2SO_4, filtration and evaporation of the solvent, the crude product was purified repeated crystallization from EtOAc/PE (5:3 v/v). Colorless solid; Yield: 70 mg (74 %). ^{1}H-NMR ($CDCl_3$, J/Hz, 400 MHz): δ = 7.44 (d, ^3J(H,H) = 8.8, 2H, Ar-H), 7.07 (dd, ^3J(H,H) = 8.2, ^4J(H,H) = 2.1, 1H, Ar-H), 7.02 (d, ^4J(H,H) = 2.1, 1H, Ar-H), 6.95 (dd, ^3J(H,H) = 8.5, ^4J(H,H) = 2.1, 3H, Ar-H), 4.21-4.01 (m, 6H, OCH, OCH_2), 3.96 (d, ^3J(H,H) = 5.2, 2H, OC**H**$_2$CH), 3.86-3.74 (m, 4H, OCH_2), 2.93 (d, ^3J(H,H) = 5.3, 1H, OH), 2.55 (d, ^3J(H,H) = 5.0, 1H, OH), 2.37 (t, ^3J(H,H) = 6.2, 1H, OH), 2.14-2.01 (m, 5H, OH, CH_2CF_2), 1.97-1.89 (m, 1H, CH), 1.73-1.59 (m, 8H, CH_2). ^{19}F-NMR ($CDCl_3$, J/Hz, 188 MHz): δ = -81.21 (t, ^3J(F,F) = 9.9, 6F, CF_3), -114.55 (s, 4F, CH_2CF_2), -122.26 (s, 12F, CF_2), -123.08 (s, 4F, CF_2), -123.89 (s, 4F, CF_2), -126.48 (s, 4F, C**F**$_2$CF$_3$). ^{13}C-NMR (Pyridin-d5, J/Hz, 100 MHz): δ = 159.1, 150.1, 149.2, 134.7, 133.7, 128.2 (2C), 119.9, 115.4, 115.4 (2C), 113.6, 72.2, 71.9, 71.5, 71.4 (2C), 71.0, 64.5, 64.3 (2C), 38.2, 31.0, 30.9, 18.0 (CH_2). calcd. for $C_{42}H_{36}F_{34}O_7$: C 38.84 %, H 2.79 %; found: 38.57 %, H 2.81 %.

2. Experimental techniques

2.1 DSC and optical microscopy

Polarized optical microscopy was carried out using Optiphot 2, Nikon in conjunction with a Mettler FP82HT heating stage). Differential scanning calorimetry was done on DSC-7, Perkin Elmer. The heating and cooling rates were 10 K min^{-1}.

2.2 X-ray scattering on powder-like and aligned samples

X-ray investigations on powder-like samples were carried out with a Guinier film camera (Huber), samples in glass capillaries (ϕ1 mm) in a temperature-controlled heating stage, quartz-monochromatized CuK_α radiation, 30 to 60 min exposure time, calibration with the powder pattern of $Pb(NO_3)_2$. Aligned samples were obtained on a glass plate. Alignment was achieved upon slow cooling (rate: 1 K·min^{-1} – 0.01 K·min^{-1}) of a small droplet of the sample and takes place at the sample–glass or at the sample–air interface, with domains fiber-like disordered around an axis perpendicular to the interface. The aligned samples were held on a temperature-controlled heating stage and the diffraction patterns were recorded with a 2D detector (HI-STAR, Siemens).

2.3 Synchrotron X-ray diffraction and electron density reconstruction

High-resolution small-angle powder diffraction experiments were recorded on Beamline I22 at Diamond Light Source. Samples were held in evacuated 1 mm capillaries. A modified Linkam hot stage with a thermal stability within 0.2 ºC was used, with a hole for the capillary drilled through the silver heating block and mica windows attached to it on each side. A MarCCD detector was used. q calibration and linearization were verified using several orders of layer

reflections from silver behemate and a series of *n*-alkanes. The measurement of the positions and intensities of the diffraction peaks is carried out using Galactic PeakSolve^{TM} program, where experimental diffractograms are fitted using Gaussian shaped peaks. The diffraction peaks are indexed on the basis of their peak positions, and the lattice parameters and the space groups are subsequently determined. Once the diffraction intensities are measured and the corresponding space group determined, 3-d electron density maps can be reconstructed, on the basis of the general formula

$$E(xyz) = \Sigma_{hkl} F(hkl) \exp[i2\pi(hx+ky+lz)] \quad \text{(Eqn. 1)}$$

Here $F(hkl)$ is the structure factor of a diffraction peak with index (hkl). It is normally a complex number and the experimentally observed diffraction intensity

$$I(hkl) = \mathrm{K}\cdot F(hkl)\cdot F^*(hkl) = \mathrm{K}\cdot|F(hkl)|^2 \quad \text{(Eqn. 2)}$$

Here K is a constant related to the sample volume, incident beam intensity etc. In this paper we are only interested in the relative electron densities, hence this constant is simply taken to be 1. Thus the electron density

$$E(xyz) = \Sigma_{hkl} \mathrm{sqrt}[I(hkl)] \exp[i2\pi(hx+ky+lz)+\phi_{hkl}] \quad \text{(Eqn. 3)}$$

for 2D structures $I(hk)$ and Eqn. (4) were used:

$$E(xy) = \Sigma_{hk} \mathrm{sqrt}[I(hk)] \exp[i2\pi(hx+ky) +\phi_{hk}] \quad \text{(Eqn. 4)}$$

As the observed diffraction intensity $I(hkl)$ is only related to the amplitude of the structure factor $|F(hkl)|$, the information about the phase of $F(hkl)$, ϕ_{hkl}, can not be determined directly from experiment. However, the problem is simplified considerably when the structure of the ordered phase is centrosymmetric; then the structure factor $F(hkl)$ becomes and ϕ_{hkl} is either 0 or π.

This makes it possible for a trial-and-error approach, where candidate electron density maps are reconstructed for all possible phase combinations, and the "correct" phase combination is then selected on the merit of the maps, helped by prior physical and chemical knowledge of the system. This is especially useful for the study of nanostructures, where normally only a limited number of diffraction peaks are observed. We have used volume *vs* electron density histograms as a guidance.[S3]

3. Additional data

3.1 DSC traces

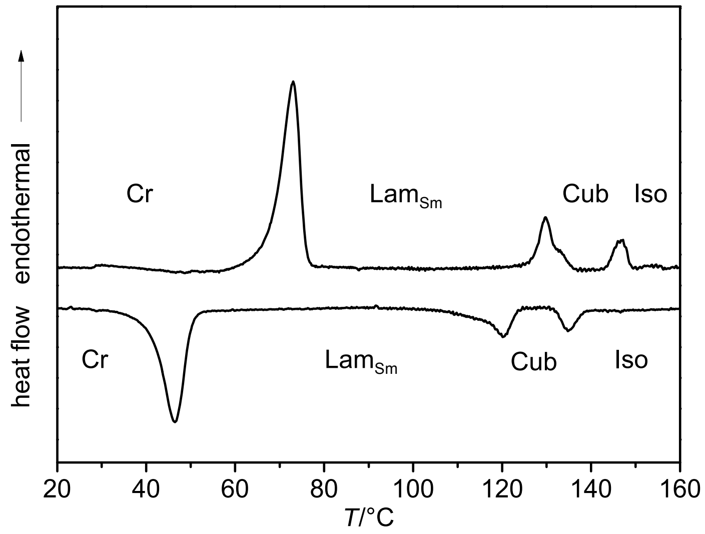

Figure S1. Compound **1:** DSC heating (top) and cooling (bottom) traces (10 K min^{-1}).

3.2 Additional XRD data

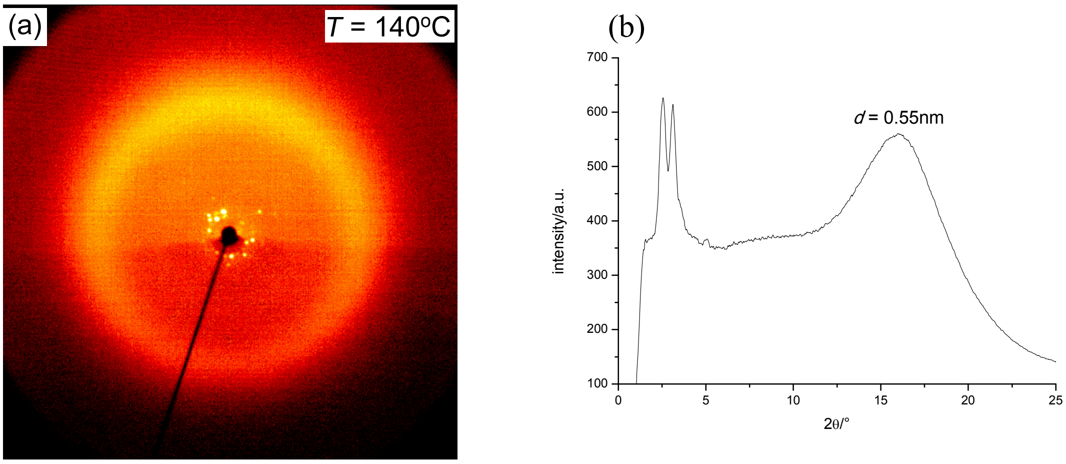

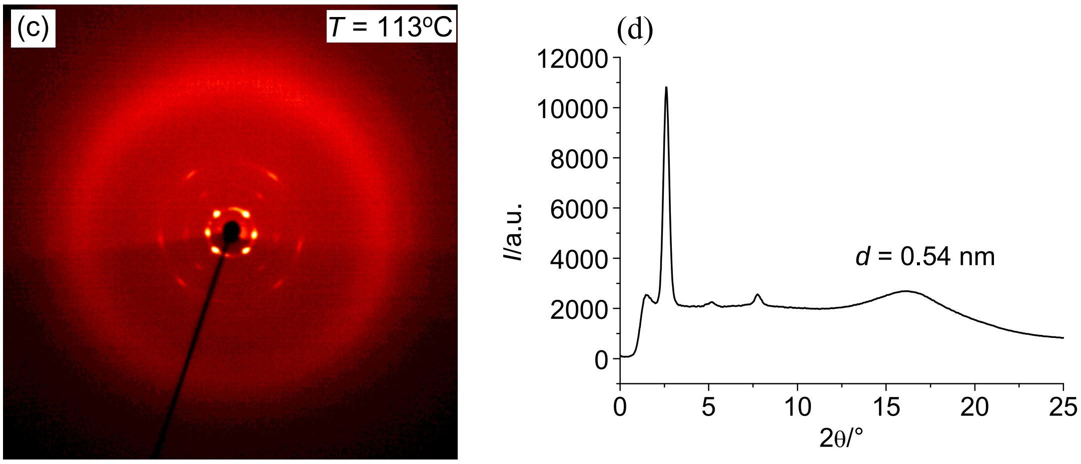

Figure S2. Compound **1:** (a) X-ray diffraction of the Cub/ $Pn\bar{3}m$ phase at 140 °C; (b) θ-scan of the wide angle region. (c) X-ray diffraction pattern of Lam$_{Sm}$ phase at 113 °C; (d) θ-scan of the wide angle region; *d* values corresponding to the maximum of the diffuse outer peak are marked in (b) and (d).

4. Synchrotron X-ray and structural data

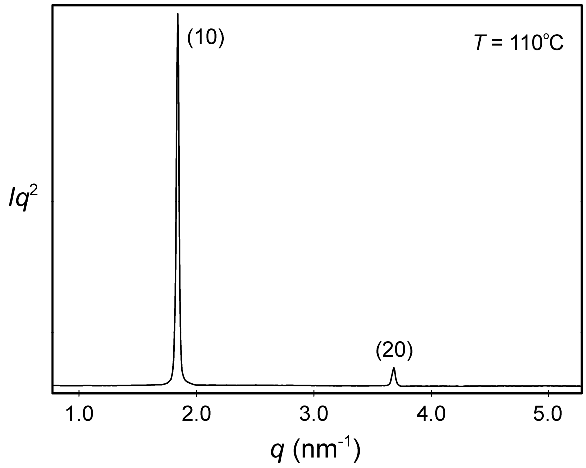

Figure S3. SAXS pattern of the Lam phase of compound **1**

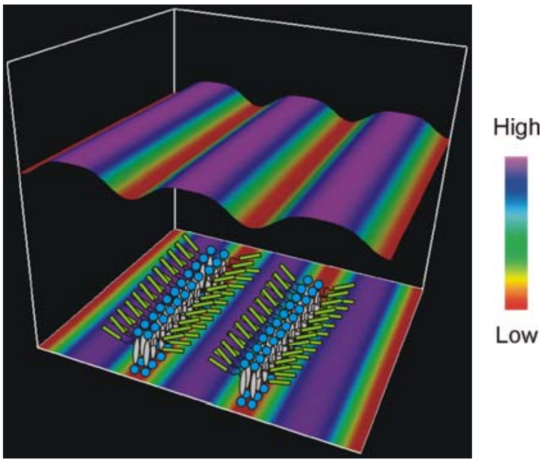

Figure S4. The Lam phase of compound **1**. The organisation of molecules is shown schematically above the color map (white: aromatic cores, blue: glycerol groups, green: perfluorinated chain ends.

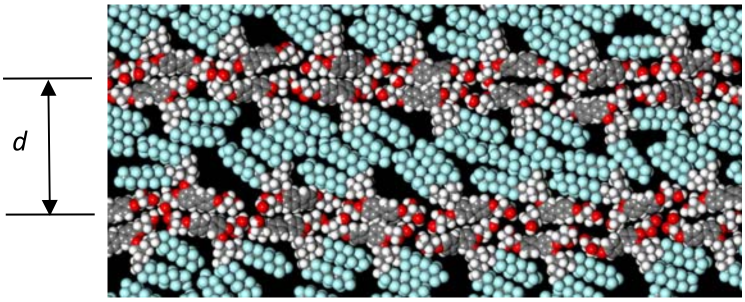

Figure S5. Molecular model of the Lam phase of compound **1** after molecular dynamics annealing simulation using Forcite module of Materials Studio (Accelrys). The layers are viewed edge-on. *d* is the layer spacing, fixed at the experimental value.

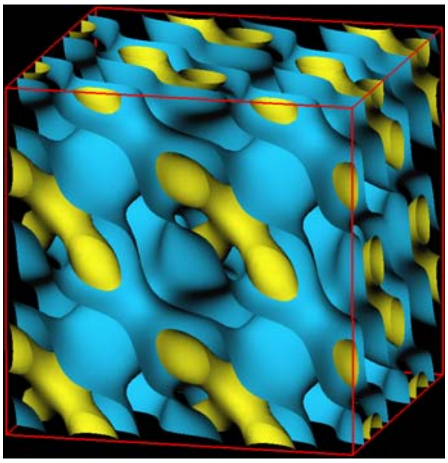

Figure S6. 3D two-colour contour electron density map of Cub/$Pn\bar{3}m$ phase of compound **1.** the The blue isoelectron surfaces enclose the perfluorinated chains (high electron density) and the yellow surfaces enclose the aromatic cores and glycerol groups (low electron density).

Table S1. Experimental and calculated *d*-spacings, relative integrated intensities, and phases used in the reconstruction of electron densities for the Lam phase of compound **1** at 110 ºC. The intensity values are Lorentz and multiplicity corrected.

(*hk*)	$d_{obs.}$ – spacing (nm)	$d_{cal.}$ – spacing (nm)	*intensity*	*phase*
(10)	3.40	3.40	100.0	π
(20)	1.70	1.70	5.5	π
d = 3.40 nm				

Table S2. Experimental and calculated *d*-spacings, relative integrated intensities, and phases used in the reconstruction of electron densities for the Cub/$Pn\bar{3}m$ phase of compound **1** at 140 ºC. All intensity values are Lorentz and multiplicity corrected.

(*hkl*)	$d_{obs.}$ – spacing (nm)	$d_{cal.}$ – spacing (nm)	*intensity*	*phase*
(110)	3.55	3.55	64.8	0
(111)	2.90	2.90	100.0	0
(200)	2.51	2.51	17.4	π
(211)	2.05	2.05	2.5	π
(220)	1.78	1.78	5.4	π
(310)	1.59	1.59	0.9	π
(311)	1.52	1.51	0.5	0
(321)	1.34	1.34	1.0	0
a_{cub} = 5.02 nm				

5. Calculation of number of molecules per cell

Table S3. Calculation of the molecular volume (V_{mol}), volume of the hypothetical unit cells (V_{cell}) and number of molecules in these unit cells (n_{cell}) of the Cubic phases.[a]

**Compound**	**1**
a_{cub} (nm)	5.02
V_{cell} (nm^3)	126.5
V_{mol} (nm^3)	1.22
V of fluorinated chain ends (nm^3)	0.62
V of rest of molecule (nm^3)	0.60
$n_{cell,cryst} = V_{cell} / V_{mol}$	104
$n_{cell, liq}$	82
n_{cell}	93
n_{bund}	11.6

[a] V_{cell} = volume of the unit cell defined by the dimensions $a_{cub}{}^3$ a_{cub}^3; V_{mol} = volume for a single molecule as calculated using the crystal volume increments;[S4] $n_{cell, cryst}$ = number of molecules in the unit cell, calculated according to $n_{cell} = V_{cell}/V_{mol}$ (average packing coefficient in the crystal is $k = 0.7$;[S5] $n_{cell, liq}$ = number of molecules in the unit cell of an isotropic liquid with an average packing coefficient $k = 0.55$, calculated according to $n_{cell, liq} = 0.55/0.7 \times n_{cell, cryst}$; n_{cell} = number of molecules in the cell of the LC phase estimated as the average between $n_{cell, cryst}$ and $n_{cell,liq}$; n_{bund} = number of molecules in each bundle, $n_{bund} = n_{cell}/8$.

6. References

[S1] M. Prehm, C. Enders, M. Y. Anzahaee, B. Glettner, U. Baumeister, C. Tschierske, *Chem. Eur. J.* **2008**, *14*, 6352-6368.

[S2] The synthesis of the branched semiperfluoroalkylbromides **Br3/8** is described in: M. Prehm, F. Liu, X. Zeng, G. Ungar, C. Tschierske, *J. Am. Chem. Soc.* **2011**, *133* (13), 4906–4916.

[S3] V. S. K. Balagurusamy, G. Ungar, V. Percec, G. Johansson, *J. Am. Chem. Soc.,* **1997**, *119,* 1539-1555.

[S4] A. Immirzi, B. Perini, *Acta Cryst. Sect. A* **1977**, *33*, 216-218.

[S5] A. I. Kitaigorodski, in “Molekülkristalle”, Akademieverlag Berlin, 1979.
